# PB.37. Does conventional imaging accurately predict the extent of disease in women with dense breasts? If so, does this have a significant impact on patient surgical outcome?

**DOI:** 10.1186/bcr3721

**Published:** 2014-11-03

**Authors:** V Scott, S Mohammadi, D Tzias, A Wale, S Heller

**Affiliations:** 1 St George's Hospital, London, UK

## Introduction

Breast density is known to mask disease. This study evaluated accuracy of conventional imaging in predicting extent of disease with respect to breast density and surgical outcome.

## Methods

Patients treated for biopsy-proven breast cancer between 1 January 2012 and 1 January 2013 were identified. Data regarding patient demographics and lesion characteristics on imaging and on surgical pathology were collected. Mammograms were assigned BI-RADS density scores. Lesion size on imaging was compared with size at final surgical pathology. Effect of breast density on disease underestimation and on final surgical outcome was analysed with two-sided Fisher's exact test.

## Results

A total of 237 women were identified; applying exclusion criteria left a final cohort of 165 patients, median age 62 years (range 24 to 96 years). In total, 114/165 (69%) patients had nondense breasts (BI-RADS 1, 2) and 51/165 (31%) had dense breasts (BI-RADS 3, 4).

Lesion size was underestimated on mammography in 131/165 (79.4%) patients, compared with 138/165 (83.6%) on ultrasound. Mammography underestimated disease extent in 42/51 (80.8%) patients with dense breasts and in 89/114 (78.1%) with nondense breasts (*P *= 0.6776). Initially, 97 patients underwent wide local excision, while 68 had mastectomy. Twenty patients needed further surgery; 17 had re-excision and three had completion mastectomy. A total 6/51 (11.8%) patients with dense breasts required further surgery, compared with 14/114 (12.2%) with nondense breasts (*P *= 1.0).

## Conclusion

Findings show that conventional imaging underestimates lesion size when compared with final histopathology in dense and nondense breasts. However, this does not have a significant effect on surgical outcome.

